# DPP4 in Diabetes

**DOI:** 10.3389/fimmu.2015.00386

**Published:** 2015-07-27

**Authors:** Diana Röhrborn, Nina Wronkowitz, Juergen Eckel

**Affiliations:** ^1^Paul-Langerhans-Group for Integrative Physiology, German Diabetes Center, Düsseldorf, Germany

**Keywords:** CD26/DPP4, soluble DPP4, type 2 diabetes mellitus, incretins, DPP4 inhibitors/gliptins, multifunctional enzyme

## Abstract

Dipeptidyl-peptidase 4 (DPP4) is a glycoprotein of 110 kDa, which is ubiquitously expressed on the surface of a variety of cells. This exopeptidase selectively cleaves N-terminal dipeptides from a variety of substrates, including cytokines, growth factors, neuropeptides, and the incretin hormones. Expression of DPP4 is substantially dysregulated in a variety of disease states including inflammation, cancer, obesity, and diabetes. Since the incretin hormones, glucagon-like peptide-1 and glucose-dependent insulinotropic polypeptide (GIP), are major regulators of post-prandial insulin secretion, inhibition of DPP4 by the gliptin family of drugs has gained considerable interest for the therapy of type 2 diabetic patients. In this review, we summarize the current knowledge on the DPP4–incretin axis and evaluate most recent findings on DPP4 inhibitors. Furthermore, DPP4 as a type II transmembrane protein is also known to be cleaved from the cell membrane involving different metalloproteases in a cell-type-specific manner. Circulating, soluble DPP4 has been identified as a new adipokine, which exerts both para- and endocrine effects. Recently, a novel receptor for soluble DPP4 has been identified, and data are accumulating that the adipokine-related effects of DPP4 may play an important role in the pathogenesis of cardiovascular disease. Importantly, circulating DPP4 is augmented in obese and type 2 diabetic subjects, and it may represent a molecular link between obesity and vascular dysfunction. A critical evaluation of the impact of circulating DPP4 is presented, and the potential role of DPP4 inhibition at this level is also discussed.

## Introduction

Dipeptidyl-peptidase (DPP) 4, which is also known as CD26, is a ubiquitously expressed glycoprotein of 110 kDa, which was first characterized by Hopsu-Havu and Glenner (1). DPP4 is a type II transmembrane protein, which is also cleaved off the membrane and released into the circulation by a process called shedding (2, 3). The importance of DPP4 for the scientific and medical community raised substantially since the approval of DPP4 inhibitors for the treatment of type 2 diabetes mellitus (T2DM). These so-called gliptins increase the incretin levels and therefore prolong the post-prandial insulin action. Since soluble DPP4 is characterized as an adipokine (4) and also correlates with parameters of the metabolic syndrome (5), it might also be an important molecular biomarker. DPP4 is a multifunctional enzyme, which serves as a binding partner for numerous peptides, among which are adenosine deaminase (ADA) and extracellular matrix proteins (2, 6, 7). Moreover, as a serine protease, DPP4 cleaves numerous substrates, which further amplifies its complexity of action. Thus, DPP4 is involved in signaling processes, immune cell activation, and its dysregulated expression and release is associated with numerous diseases.

In the present review, we wanted to emphasize the complex function of DPP4 with special focus on its association to T2DM. Furthermore, we wanted to offer a different perspective of the current view of DPP4 beyond the inhibition of its protease activity (8–10). The first part of the present review is dealing with general information about DPP4 and its numerous biological functions in regard to T2DM and its treatment. The last section collects the current knowledge about how DPP4 with its pleiotropic functions, as described before, affects several organs, thereby playing a pivotal role in the development of T2DM and its comorbidities.

## General Information on DPP4

### Biology of DPP4

The following part will deal with the domain architecture and respective relevance of these domains for the functionality of DPP4.

**Figure 1:**
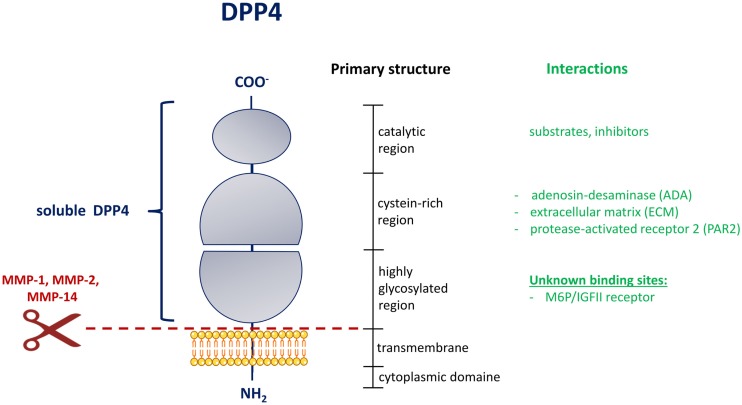
**Domain structure of DPP4 [adapted from Ref. (2)]**. Schematic representation of the membrane-bound DPP4 monomer. The extent of the circulating and soluble form of DPP4 is illustrated on the left in blue. The shedding of DPP4 from the membrane by indicated matrix metalloproteinases is shown by a scissor symbol in red. The vertical black bar on the right represents the primary structure with the delineation of the different regions. In green are interactions collected, which occur in the indicated region of the DPP4 structure. MMP, matrix metalloproteinase; M6P/IGFII, mannose-6 phosphate/insulin-like growth factor 2.

Dipeptidyl-peptidase 4 (EC3.4.14.5) is a type II transmembrane protein, which groups together with fibroblast-activation protein α (FAP), the resident cytoplasmic proteins, DPP8 and DPP9, and the non-enzymatic members, DPP6 and DPP10, to the serine peptidase subfamily S9B. All of these proteins share a typical α/β hydrolase fold (2, 6). The DPP4 protein consists mainly of 4 domains: a short cytoplasmic domain (1–6), a transmembrane domain (TMD) (7–28), a flexible stalk segment (29–39), and the extracellular domain (40–766), which can be further separated by a highly glycosylated region, the cysteine-rich region, and the catalytic region (Figure 1).

As a member of the type II transmembrane proteins, DPP4 contains a typical signal peptide, which is necessary for the targeting to the endoplasmatic reticulum and the initiation of the translocation across the cell membrane. In contrast to the classically secreted proteins, the signal peptide is not cleaved off, but serves as a membrane anchor. We were able to show that the circulating form of DPP4 (sDPP4), which lacks the cytoplasmic domain and the transmembrane region, is cleaved off the membrane of human adipocytes and smooth muscle cells in a process called shedding by the involvement of matrix metalloproteases (MMPs) (3).

Within the TMD, it could be shown that proline residues play an important role for the translocation of membrane-anchored proteins, such as DPP4. Chung and colleagues (11) studied single proline substitution throughout the TMD of DPP4. They were able to show that translocation and integration into the membrane are determined by the hydrophobicity, conformation, and also the location of proline within the TMD. Furthermore, the position of proline relative to other prolines and the location of highly hydrophobic residues within the TMD are important for correct translocation and membrane integration of DPP4.

In addition to the TMD, the glycosylation of DPP4 is also important for the correct trafficking of DPP4. Carbohydrates account for approximately 20% of the total molecular mass of DPP4 and cause heterogeneity of this protein depending on the location on different cell types. Two highly conserved glutamate residues (205 and 206) within the glycosylated region are essential for the activity of DPP4 (12). Interestingly, six of the nine N-glycosylation sites are located within the glycosylated region. These glycosylation sites are mostly conserved among species. They are necessary for folding, stability, and intracellular trafficking (13). Other modifications like sialylation and/or O-glycosylation have an impact on targeting DPP4 to the cell membrane. Sialyation of DPP4 increases significantly with age, and hypersialyation occurs in patients with HIV infection (14).

Not only glycosylation and residues within the TMD are important for the cellular function of DPP4 but also dimerization. DPP4 can be found as monomer, homodimer, or even as homotetramer on the cell surface of cells. DPP4 needs dimerization for enzymatic activity, and this is the predominant form of DPP4 (15). Dimerization occurs upon interaction with DPP4 itself or with other binding partners, e.g., FAP (16, 17), and occurs via interaction with the cysteine-rich region. Through its interaction with several proteins, DPP4 can act also in an enzymatic activity-independent way. Through this interaction, DPP4 is linked to various mechanisms like immune response and tumor invasion. The heterodimerization and interaction with different binding partners will be discussed in a later section.

The serine in the active site of DPP4 is located in the sequence Gly–Trp–Ser–Tyr–Gly and is part of the catalytic triad (Ser 630, Asp 708, His 740) within the catalytic region of DPP4. DPP4 is an exopeptidase, which cleaves dipeptides from the penultimate N-terminal position of its substrates and thereby either inactivates these peptides and/or generates new bioactive compounds (7). There are numerous different DPP4 substrates known to date and they will be addressed in a separate section within this review.

### DPP4 expression and its regulation

Dipeptidyl-peptidase 4 is ubiquitously expressed on numerous different cell types among which are epithelial cells, fibroblasts, and leukocyte subsets. Mechanisms that regulate DPP4 gene transcription and enzymatic activity are not fully understood so far and may be dependent on the studied cell type.

The human DPP4 gene is located on chromosome 2, spans 70 kb, and consists of 26 exons (2). The DPP4 promoter region contains consensus sites for different transcription factors like NFκB, SP-1, EGFR, and AP-1 factor NF-1 (18). At least in chronic b lymphocytic leukemia cells, it could be shown that there is a consensus interferon γ-activated sequence (GAS), which is a binding motif for STAT1. The interferons, α, β, and γ, stimulate STAT1α binding to this region and thus lead to an increased DPP4 expression and activity (19). Interleukin (IL) 12, which is a key factor in differentiation of naïve T-cells into the Th1 subtype, is also able to upregulate DPP4 expression. Therefore, DPP4 is important in immune cell activation (20, 21). Our group was able to show that release of soluble DPP4 is increased upon TNFα stimulation and insulin *in vitro* (4). However, IL-12 and TNFα also seem to play a regulatory role in translation and translocation of DPP4. In activated lymphocytes, IL-12 upregulates DPP4 translation whereas TNFα decreases cell surface expression, which might be due to elevated sDPP4 release (22). Also transcription factors, such as HIF-1α and HNFs, target DPP4 expression (23), which fits to the observation of our group that hypoxia induces DPP4 release in human smooth muscle cells, which might be mediated by MMPs (3).

### Non-enzymatic interactions of DPP4

Through its cysteine-rich region, which is separated from the catalytic region, DPP4 is able to interact with different proteins, and further broadens its spectrum of activity and highlights its multifunctional role in different processes.

#### Binding Partners of Membrane-Bound DPP4

The best-studied interaction in this regard is certainly the binding of DPP4 and ADA. It was already identified in 1993 by Morrison and colleagues (24). Importantly, the interaction of DPP4 and ADA preserves the enzymatic function of both binding partners. It has been shown that residues 340–343 of DPP4 are essential for the interaction with ADA. Regulation of the DPP4/ADA interaction occurs, e.g., via tetramerization of DPP4 or glycosylation at Asn281, which interferes with ADA binding (25). Also, the HIV envelope glycoprotein, gp120, which interacts with DPP4 on lymphocytes via its C3 region, is able to inhibit the association with ADA (2). Upon ADA binding, activation of plasminogen-2 occurs, which raises plasmin levels. This leads to a degradation of matrix proteins and an activation of MMP, thereby indicating that the interaction of DPP4 and ADA might be involved in tissue remodeling (26).

Furthermore, ADA catalyzes the irreversible deamination of adenosine and 2′-deoxyadenosine and is therefore a crucial player in the cellular and humoral immunity. Via interaction with CD45, the complex of ADA and DPP4 enhances T-cell activation. Interestingly, DPP4 is also able to promote T-cell proliferation independent from ADA binding or even its enzymatic activity (27). Zhong et al. were able to show that the interaction of DPP4 and ADA on dendritic cells might potentiate inflammation in obesity upon activation and proliferation of T-cells, which could be competitively inhibited by exogenous sDPP4, but not by inhibiting DPP4 enzymatic function (28). Furthermore, ADA activity is elevated in T2DM patients and may serve as a marker of inflammation and obesity (29).

Beside its role in inflammation, adenosine is also an important player in glucose homeostasis. Already in 1988 it was shown that, by lowering endogenous adenosine levels, ADA contributes to a reduced insulin sensitivity of glucose transport stimulation (30). Additionally, adenosine seems to facilitate insulin action in adipocytes (31). Another study could show a correlation of increased ADA activity in T2DM with fasting plasma glucose, HbA1c, aspartate, and alanine aminotransferase (ALT). DPP4 inhibitors exert no additional effects on ADA activity despite glycemic control or HbA1c-dependent effects (32). All these studies emphasize that the effects of ADA/DPP4-interaction are independent of DPP4 enzymatic activity.

Another known interaction partner of DPP4 is Caveolin-1, which is present on antigen-presenting cells (APCs) and binds to residues 630 and 201–211 of DPP4 expressed on T-cells. Thereby, an upregulation of CD68 occurs and initiates a signaling cascade, which might be implicated in the pathogenesis of arthritis, and may be relevant for other inflammatory diseases as well (33). Intracellular signaling is also initiated by DPP4 via interaction with Caspase recruitment domain containing protein 11 (CARMA-1) (6).

Another well-known interaction of DPP4 is with extracellular matrix proteins like collagen and fibronectin (34, 35). The interaction of DPP4 with fibronectin was revealed via nitrocellulose binding assays in rat hepatocytes and seems to play a role in the interaction of these cells with the ECM and with matrix assembly (36). Interaction of DPP4 with FAPα leads to a local degradation of ECM and thus migration and invasion of endothelial cells (37).

#### Potential Receptors for sDPP4

Since DPP4 is shedded from the membrane of cells with intact enzymatic and cysteine-rich region, it can also exert biological functions in a paracrine or endocrine manner. These functions might also involve intracellular signaling events in the targeted cells. Therefore, it would be of great importance to know receptors of sDPP4 to better understand the multiple role of sDPP4 on different cells and in different disease conditions where serum levels are elevated. However, there is not much known about DPP4 receptors so far.

Ikushima et al. were able to show that DPP4 needs to associate with mannose-6 phosphate/IGF-IIR to exhibit its function as T-cell activator. This is due to the fact that for this activation, internalization of DPP4 is necessary, but DPP4 lacks a signal for exocytosis. The binding with M6P/IGF-IIR occurs via M6P residues in the carbohydrate moiety of DPP4 and the complex is then internalized and able to exert its biological function (38).

Our group showed that at least in human vascular smooth muscle cells, protease-activated receptor 2 (PAR2) might be activated by sDPP4. We were able to show that sDPP4-mediated ERK activation and proliferation, as well as upregulation of inflammatory cytokines could be prevented by silencing of PAR2. The same was shown by use of a specific PAR2 antagonist. We propose that sDPP4 acts as an activator of PAR2, since a sequence within the cysteine-rich region of DPP4 is highly homologous to the auto-activating tethered ligand of PAR2 (39).

### Genetic alterations of DPP4 and predisposition to T2DM-associated diseases

There are only few studies aiming to identify modifications in the DPP4 gene and their association with T2DM. Some of these are reviewed in the following section.

In 2009, Bouchard et al. analyzed single nucleotide polymorphisms (SNPs) in the DPP4 gene and searched for association with blood pressure, lipids, and diabetes-related phenotypes in obese individuals, to verify whether DPP4 gene polymorphisms could explain the individual risks of obese patients to develop metabolic complications. Three of the analyzed SNPs showed significant association with plasma total-cholesterol levels or plasma triglyceride level or total cholesterol level. But none of the polymorphisms or cardiovascular disease risk factors showed a significant correlation with DPP4 mRNA levels in omental adipose tissue. Therefore, the authors concluded that, at least in their studied group, DPP4 gene polymorphisms seem to be unrelated to the inter-individual risk of developing obesity-related metabolic complications (40).

In another study, visceral adipose tissue DNA of 92 severely obese, non-diabetic female patients was analyzed for methylation rate in the DPP4-promoter CpG island and compared between different DPP4 polymorphisms. These cytosine- and guanine-rich regions are prone to epigenetic modification like methylation, and thus inactivate or activate transcription of certain genes. Different methylation levels of the DPP4 gene were identified in three DPP4 SNPs. Interestingly, the methylation level was negatively associated with DPP4 mRNA abundance and positively with plasma total/HDL-cholesterol ratio. These observations suggest that plasma lipid profile is improved by a higher methylation status of the investigated CpGs (41). Two years later, the same group analyzed DPP4 gene methylation levels between obese subjects with and without the metabolic syndrome in visceral adipose tissue. They observed no significant difference in the percentage of methylation levels of the CpGs within or near the second exon of the DPP4 gene between non-diabetic severely obese subjects with or without metabolic syndrome. However, they were able to show a correlation between plasma cholesterol levels and the percentage of methylation when the subjects were classified into quartiles (42). This further underpins a link between epigenetic modification of the DPP4 gene and plasma lipid metabolism.

Aghili et al. analyzed 875 patients with angiographically documented coronary artery disease (CAD), and divided them in two subgroups dependent of their myocardial infarction (MI) status. By a genome-wide association study, loci, which predispose to MI, were assessed and associated with SNPs in the DPP4 gene. They found that polymorphisms in the DPP4 gene increase the risk of MI and progression of atherosclerosis in terms of plaque stability in patients with already existing CAD. Especially, one SNP was identified in both dominant and additive inheritance modes, which associates with low plasma DPP4 levels and which may increase the risk of MI in CAD patients (43).

Dyslipidemia, which is characterized by excessive lipids in the blood, is a common feature of T2DM. The status of this risk factor is quantifiable by the measurement of apolipoprotein B (ApoB) in the blood. In a very recent study by Baileys and colleagues, they aimed to identify novel SNPs associated with ApoB level. Especially in South Asians, who tend to develop risk factors for T2DM and MI at younger ages and lower BMI, they found an association of a DPP4 SNP with ApoB level (44).

## The DPP4 Deficiency in Animal Models

To date, there are several studies dealing with the question, which role DPP4 plays *in vivo*. Animal models are useful tools to study the involvement of DPP4 in different organs. Upon triggering different diseases like insulin resistance (IR) or MI, it is possible to understand the role of DPP4 in these comorbidities of T2DM.

### DPP4 deficiency in rats

A major part of the literature is dealing rather with DPP4-KO in rats than in mice. Most research groups work with the F344/DuCrj (DPP4-deficient) strain. Rats developing IR due to high-fat diet (HFD) feeding showed improved HOMA-IR values and blood glucose levels in oral glucose tolerance test (oGTT) and more active glucagon-like peptide-1 (GLP-1) and insulin in plasma (45). The same improved glucose tolerance with increased GLP-1 and leptin levels was found in DPP4-depleted Dark Agouti rats with diet-induced obesity (46). Another research group also found improvement in serum lipid profile despite increased visceral fat. They also performed insulin tolerance tests (ITT) in addition to GTT and saw an increased phosphorylation of Akt and reduced expression of gluconeogenic genes, concluding that DPP4-KO improved insulin sensitivity. Furthermore, the KO rats showed increased adipocyte maturation by increased expression of genes involved in triglyceride uptake and in PPARγ expression and increased adiponectin and leptin levels. In addition, adipose tissue is less inflamed illustrated by lower TNFα, IL-6, PAI1, and CCL7 levels (47). The observed effects were attributed to elevated glucose-dependent insulinotropic polypeptide (GIP) levels in the KO rats. Furthermore, the same group could also show attenuated liver damage under HFD challenge in the KO rats due to improved bile secretory function. They postulate that the enhanced export of bile acids out of hepatocytes and a reduction of bile acid synthesis via inhibition of CYP7A1, which converts cholesterol to bile acids, were mediated by increased GLP-1 in DPP4-KO rats (48). Interestingly, at least Yasuda and colleagues also saw a significant reduced food intake in the KO rats irrespective of the diet (45), which might be due to changed receptor specificity of neuropeptide Y (NPY), which was shown to be more potent in KO rats to influence food intake and feeding motivation (49). Although several independent working groups saw increased NPY levels in KO rats (49–51), the effect on food intake is controversial (45, 50). When diabetes is induced via streptozotocin (STZ) treatment in F344/DuCrj-DPP4-deficient rats, onset of hyperglycemia was delayed, but KO rats showed impaired creatinine clearance and more severe dyslipidemia, which might be caused by a dysregulated expression of factors involved in steroid and lipid metabolism (52, 53). The authors concluded that DPP4 might be responsible for preservation of renal function. Another effect of the whole-body KO of DPP4 in rats is induction of behavioral changes like a blunted stress phenotype (46, 51) and also effects on the immune-regulatory system like blunted NK cell and T-cell function and differential leukocyte subset composition or altered cytokine levels (46, 47).

### DPP4 deficiency in mice

Most of the observations already described in deficient rats are also true in whole-body DPP4-KO mice. Marguet et al. showed enhanced glucose tolerance, lower plasma glucose, and higher plasma insulin and GLP-1 after a 15 min oral glucose load without further characterizing the diet, age, or sex of the used C57BL/6 DPP4-KO mice (54). Conarello and colleagues found less weight-gain independent of the diet, and marked hypertrophy in the HFD-fed KO mice in epididymal white (eWAT) and brown adipose tissue (BAT). Importantly, they admitted that the reduction in caloric intake accounted for ~70% of the observed changes in bodyweight. Although they still observed differences in the bodyweight between KO and WT when they used pair-feeding, they carried out their further analysis in *ad libitum* fed mice and it is therefore difficult to judge the influence of DPP4 irrespective of bodyweight. However, they found improved insulin sensitivity and islet morphology, and improved liver biology in respect to lipid content and marker gene expression (55). The observation that DPP4 might be involved in the immune-regulatory system was also investigated in DPP4-KO mice, which were treated with pokeweed mitogen that stimulates growth and proliferation of B-cells. DPP4 seems to be involved in maturation and migration of immune cells, cytokine secretion, and percentages of spleen lymphocytes (56).

All these studies have in common that they use whole-body KO animals. The disadvantage here is that one cannot distinguish between direct effects of the KO and side effects caused, for example, by different immune cell status or decreased caloric intake. To really decipher the role of DPP4 in different tissues and their crosstalk with other target tissues, it is of great importance to study tissue-specific KO models.

Because of this and because we were the first to describe DPP4 as a novel adipokine linked to parameters of the metabolic syndrome (4), we decided to develop an adipose tissue-specific KO mouse model. The AT-specific DPP4-KO mouse was generated using a Cre-lox strategy under control of the aP2 promoter on the C57BL/6J background. Interestingly, we found out that KO mice gained significantly more weight, fat, and lean mass under HFD with no effect on energy expenditure or food intake. However, KO mice showed improved HOMA-IR and lower fasting insulin. The observations that within AT, KO mice display a shift toward significantly more smaller adipocytes, and an increased expression of M2 macrophage marker genes points toward a beneficial role of DPP4 deletion in adipose tissue remodeling during HFD (57, 58).

## Enzymatic Function of DPP4

Dipeptidyl-peptidase 4 exerts its enzymatic action by clipping dipeptides from the penultimate position of its substrates. The active center, which is housed in an internal cavity, is surrounded by the β-propeller domain and the catalytic domain. Inhibitors and substrates enter/leave the active center by a so-called “side opening” (59, 60). The following section deals with known substrates of DPP4 in respect of T2DM, and with DPP4 as a drug target for T2DM treatment, which will include current knowledge on DPP4 inhibitors and the impact of DPP4 on organs involved in complications of T2DM.

### DPP4 substrates

In theory, numerous peptides are potential DPP4 substrates since they contain the cleavable amino acid sequence at their penultimate position, but not for all of them it could be shown that DPP4 is able to cleave them *in vivo*. There seems to be a size limitation at least for cytokines, where DPP4 is more prone to cleave substrates of around 24 amino acids (aa) length. Furthermore, the substrate recognition is also dependent on the aa sequence around the penultimate position (61, 62). It turned out to be difficult to find physiological targets of DPP4 in the literature, reasons of that are excellently summarized in a recent review by Mulvihill and Drucker (6). We decided to focus here on the (potential) substrates of DPP4, which might play a role in T2DM or its complications. The list of DPP4 substrates mentioned here is not fully complete and aims to highlight the importance of DPP4 in T2DM also beyond its well-known incretin effect.

#### Incretin Hormones

The incretin hormones account for approximately 50% of the insulin secretion after meal intake and are secreted from the gut within minutes after the meal intake. Through binding to distinct receptors on beta cells in the pancreas, they stimulate insulin secretion and suppress glucagon release depending on the blood glucose level. Most potent in their glucose-lowering action are glucagon-like peptide 1 (GLP-1) and GIP. Both peptides belong to the same glucagon peptide superfamily and share significant aa character.

Glucagon-like peptide-1 is secreted from L-cells of the gut into the bloodstream. Upon binding to G-protein-coupled receptors on the beta cells, intracellular cAMP level is elevated and the protein kinases, Epac1 and 2, are activated, which leads to an increase of insulin secretion. Furthermore, GLP-1 enhances beta-cell mass by mediating proliferation and differentiation and inhibiting apoptosis (8). By inhibiting gastric emptying, GLP-1 also improves blood sugar excursion, delays food absorption, and is therefore a regulator of satiety and appetite also through the hypothalamus (63).

Glucose-dependent insulinotropic polypeptide is a 42 aa peptide, which mainly originates from enteroendocrine K cells (64). Subjects with diabetes or impaired glucose tolerance show significantly reduced levels of meal-stimulated circulating GIP and the levels are negatively correlated with the severity of IR in the patients (65, 66). GIP has, in contrast to GLP-1, no effect on glucagon secretion, but also regulates fat metabolism in adipocytes (67).

Since inhibition of DPP4 due to genetic deletion or use of DPP4 inhibitors was shown to elevate GLP-1/GIP levels in numerous studies, this effect is the main focus of developing therapeutic targets for treatment of T2DM. There are numerous reviews, which focus on DPP4- and GLP-1-mediated effects, and this topic will not be further discussed here.

#### Stromal Cell-Derived Factor-1α/CXCL12

Stromal cell-derived factor-1 (SDF-1) is a chemokine that promotes angiogenesis and attracts endothelial progenitor cells (EPC) by binding to its receptor C–X–C motif chemokine receptor type 4 (CXCR4). SDF-1 is thus discussed in the literature as important mediator of cardioprotective effects addressed to the use of DPP4 inhibitors (further discussed in “DPP4 Substrates: SDF-1- and BNP-Dependent Effects of DPP4 Inhibitors”). It is a well-known physiological target of DPP4 (68, 69). SDF-1α also plays a role in diabetes itself by protecting stem-cell-derived insulin-producing cells from glucotoxicity under high glucose conditions (70) or promoting pancreatic beta-cell survival in mice via Akt activation (71). Furthermore, it was shown that some genetic variants of SDF-1α are associated with late stage complications in T2DM patients (72, 73).

#### NPY and PYY

Neuropeptide Y and peptide YY are members of the polypeptide family. They are highly expressed in the hypothalamus but are also present in peripheral tissues like islets. NPY regulates energy balance, memory, and learning, while PYY reduces appetite, inhibits gastric motility, and increases water and electrolyte absorption in the colon (74). Both NPY and PYY play a role in beta-cell survival and in glucose homeostasis (74). NPY is able to suppress insulin secretion acutely (75). Both polypeptides have in common that DPP4 truncation shifts their receptor specificity and thus alters their biological role in different cellular processes. *In vitro* experiments in adipocytes could show that DPP4 inhibition has an impact on lipid metabolism mediated by NPY (76, 77).

#### Substance P

Substance P is a physiological target of DPP4, which is sequentially converted to SP (3–11) and SP (5–11) *in vivo* in F344–DPP4-positive rats (78). SP is a neurotransmitter and modulator, which is involved in neurogenic inflammation. Serum levels in diabetes are controversially discussed with one study showing a decrease in diabetic patients (79), and another one showing an increase in fasting blood samples with correlation to diabetic risk factors like BMI and blood pressure (80). This discrepancy in serum levels could be addressed to the fact that it is not always stated which form of SP (full length versus truncated) is measured. However, SP was shown to promote IR *in vitro* in human preadipocytes by interacting with proteins that are involved in the inhibitory phosphorylation of IRS-1. Furthermore, SP can directly inhibit insulin-dependent glucose metabolism in rat adipocytes (81). SP also promotes diabetic corneal wound healing, as shown by Yang and colleagues (82).

#### Brain Natriuretic Peptide

Brain natriuretic peptide is responsible for vasodilation, natriuresis, and suppresses renin secretion. It is so far only a predicted DPP4 substrate, which was cleaved *in vitro* by DPP4 to BNP (3–32). This truncation was inhibited by a DPP4 inhibitor in a dose-dependent manner (83). Truncated forms of BNP with lower enzymatic activity are discussed as an indicator of heart failure severity. In 2013, dos Santos et al. could show an improved cardiac performance in sitagliptin-treated rats, which they attributed to increased levels of active BNP (84).

#### Pituitary Adenylate Cyclase-Activating Polypeptide

Pituitary adenylate cyclase-activating polypeptide (PACAP) is very rapidly degraded by DPP4 to the fragments (3–27), (5–27), and (6–27). These fragments lack PACAPs insulinotropic ability, but are no feasible treatment options for T2DM because of their actions on glucose homeostasis and glucagon secretion (85). Several studies have shown that PACAP is a powerful stimulator of insulin secretion, which enhances glucose uptake in adipocytes and augments antilipolytic action of insulin (86, 87). After DPP4-inhibitor treatment in mice, PACAP-induced insulin secretion was enhanced (88). However, a proof that PACAP also plays a role in humans is lacking so far.

#### Regulated on Activation, Normal T-Cell Expressed and Secreted/CCL5

Regulated on activation, normal T-cell expressed and secreted (RANTES) recruits leukocytes into inflammatory sites and is cleaved by DPP4 to RANTES (3–68). Due to this truncation, RANTES (3–68) is not able anymore to increase cytosolic calcium concentrations and to induce chemotaxis of human monocytes *in vitro*. This is explained by a shift in receptor subtype-specificity toward enhanced activation of CC-motif-chemokin-receptor 5 (CCR5) (89). Elevated serum levels of RANTES in T2DM are associated with post-prandial hyperglycemia (90). Interestingly, RANTES and its receptor CCR5 are important mediators of obesity-induced inflammation, which was shown in CCR5-KO mice (91). Levels of RANTES and CCR5 were reduced in adipose tissue of obese patients upon exercise (92). RANTES reduces glucose-stimulated GLP-1 secretion *in vitro* and *in vivo* in mice, by acting most probably through the intestinal glucose transporter SGLT1 (93).

#### Eotaxin/CCL11

Eotaxin mediates mobilization of eosinophils into the bloodstream, which was shown to be increased in DPP4-deficient F344 rats (94). DPP4 cleaves eotaxin to eotaxin (3–74). However, there was no significant correlation of eotaxin seen in patients with T2DM or impaired glucose tolerance in the KORA cohort (95).

### DPP4 as a drug target for the treatment of T2DM

#### Deactivation of DPP4 Enzymatic Activity

##### DPP4 inhibitors

Major DPP4 substrates are the so-called incretin hormones, which are key regulators of post-prandial insulin release. DPP4 inhibition leads to greater bioavailability of these proteins and therefore prolongs the half-life of insulin action. The majority of effects seen upon DPP4-inhibitor treatment are ascribed to an increase in GLP-1 levels. Because of this, DPP4 became a major target for the treatment of T2DM. This section deals with the most recent knowledge around DPP4 inhibitors, their mode of action – if known – and the newest developments in the inhibition of DPP4 enzymatic activity. There are numerous modifications and potential optimizations of the five so far approved gliptins reported. However, most of them are not in clinical trials yet and not much is known about their advantage in a head to head comparison to established gliptins. Therefore, we decided to focus on the most recent data on approved gliptins in this review. The data are also summarized in Table 1.

**Table 1:** **Summarized properties of gliptins**.

Inhibitor	Approved since	Binding mode	Kind of inhibition	Route of excretion	IC50 value	Reference
Sitagliptin	2006 FDA	S1, S2, and S2 extensive subsites	Competitive inhibition	Mostly renal route	19 nM	(10, 98–100)
Vildagliptin	2007 European medicines agency	Only S1 and S2 subsite	Substrate–enzyme blocker	Mostly renal route	62 nM	(10, 209)
Saxagliptin	2009 FDA	Only S1 and S2 subsite	Substrate–enzyme blocker	Mostly renal route	50 nM	(10, 209)
Linagliptin	2011 FDA	S1, S2, and S1′ subsites	–	Through biliary route	1 nM	(10)
Alogliptin	2013 FDA	S1, S2, and S1′ subsites	Competitive inhibition	Mostly renal route	24 nM	(8, 10)
Teneligliptin	2012 Japan	S1, S2, and S2 extensive subsites	Very potent because of unique anchor-lock domain and J-shape of molecule	Mostly renal route	0.37 nM	(97)
	2014 Korea	

Dipeptidyl-peptidase 4 inhibitors lower DPP4 activity by 70–90%. They do not pass the blood–brain barrier and have no direct effect on satiety or on altering gastric emptying (8). The benefit for diabetes therapy clearly is their indifference on bodyweight gain and the low risk of hypoglycemia. There are five gliptins approved so far for clinical use, namely sitagliptin, vildagliptin, saxagliptin, linagliptin, and alogliptin. Three more gliptins, teneligliptin, anagliptin, and trelagliptin are only approved in the Japanese and Korean market. Despite the same mode of action, the different gliptins diverge in their pharmacodynamic and pharmacokinetic properties, which might be clinically relevant for some patients (9, 96). The peptide mimetic compounds, vilda-, saxa, and teneligliptin, were identified by replacement experiments of peptide-based substrates, whereas the non-peptide mimetic compounds, sita, alo-, and linagliptin, were derived from initially found inhibitors of random screenings. The diverse chemical structures also explain the unique binding modes of the inhibitors to DPP4 (10).

The six inhibitors have been classified into three classes depending on their different binding modes in the DPP4 active center (10). Class 1 contains vilda- and saxagliptin, which only bind to the S1 and S2 subsites and form a covalent bond with the nitrile group of their cyanopyrrolidine moiety and Ser630 of DPP4. Saxagliptin has a fivefold higher activity in blocking DPP4 than vildagliptin. Group 2 contains alo- and linagliptin, which also interact with the S1′ subsite or even in case of linagliptin with the S2′ subsite. The uracil rings of both gliptins induce a conformational change in the Tyr547 of the S1′ subsite. Because of the additional interaction of linagliptin with S2′ subsite, it has an eightfold higher activity than alogliptin. The third class has the highest inhibitory function toward DPP4, because both sita- and teneligliptin interact with the S2-extensive subsite of the DPP4 active center, and an increasing number of interactions seems to increase the potency of the gliptin (10). Teneligliptin, which is only approved for T2DM treatment in the Japanese and Korean market so far, also has a unique structure characterized by a J-shape and an anchor-lock domain, which explains the strong inhibitory function and the low IC_50_ value of this drug [for review, see Ref. (97)]. The binding of the DPP4 S2-extensive subsite of some inhibitors also guarantees a high specificity toward DPP4 since other close-related peptidases like DPP8, DPP9, and FAP lack this subsite. All DPP4 inhibitors have in common that they build salt bridges with Glu-residues in the S2 subsite (10). At least for sitagliptin, it is also known that it lowers the level of free fatty acids (FFA) and thereby also comprises insulin-sensitizing properties (98). Furthermore, sitagliptin was shown to have potent anti-inflammatory properties by suppressing expression of pro-inflammatory genes in mouse and humans (98, 99). In patients with renal impairment, which is a very common complication of T2DM, sitagliptin is more suitable than sulfonylureas (100). Anagliptin, which is only approved since 2012 in the Japanese market, seems to have serum lipid-lowering and anti-atherogenic actions as well, which makes it unique among the gliptins approved so far (101, 102). Anagliptin has an IC50 of 3.3 nM and its main excretion route is renal elimination (101, 103). However, since this gliptin is only on the market since 2012, comparative head-to-head trials and data on the long-term use are missing at the moment. Shinjo and colleagues demonstrated that anagliptin exerts anti-inflammatory effects on macrophages and adipocytes *in vitro* and on inflamed mouse livers *in vivo*. In this very recent study, anagliptin was more potent in its anti-inflammatory actions than sitagliptin (104). To improve the quality of life of T2DM patients, development of novel agents now is more and more focused on long-acting agents. There are two more gliptins, which only have to be applied once weekly, namely SYR-472 (trelagliptin) and MK-3102 (omarigliptin) (105, 106). Trelagliptin is approved in Japan since 2015.

Although some authors claim that DPP4 inhibitors are only beneficial in early stages of diabetes, this could be rebutted by the work of Kumar and Gupta (107). They could show beneficial effects of three gliptins (sita-, saxa-, and vildagliptin) in lowering HbA1c also in patients with longstanding T2DM for more than 10 years. Thus, DPP4 inhibition also plays an important role irrespective of the duration of diabetes.

What has to be mentioned in respect of the beneficial roles of DPP4 inhibitors is that more and more studies about their beneficial pleiotropic effects are upcoming, which are also discussed in the following section of this review dealing with different organs. There are reports that gliptins themselves have effects on lipid profile and blood pressure as well as on inflammatory processes (108). In addition to the incretins, there are some DPP4 substrates, like SDF-1α, which might explain potential cardioprotective effects, which are discussed for gliptins. However, cardiovascular outcomes are still widely debated and controversially evidenced. Ongoing long-term studies will further shed light on the respective role of DPP4i beyond glucose homeostasis. Furthermore, one has to keep in mind that also DPP4 has direct effects independent of its enzymatic activity, like activation of downstream signaling events upon receptor binding, which are not well understood so far. Which role DPP4 inhibition plays on T2D relevant organs/comorbidities will be the topic of the following sections.

##### Alternative modes of DPP4 inhibition

Very recently, Pang and colleagues published a different strategy to inhibit DPP4 activity. They used DPP4-targeted immune therapy by vaccines in a C57BL/6J mouse model and were able to show comparable effects like in treatments with gliptins regarding GLP-1 plasma levels and post-prandial glucose excursion and insulin sensitivity in HFD-fed mice. Furthermore, they observed no side effects on immune cell activation by the DPP4 vaccine. An advantage of this method is the long-lasting effect of the vaccine in the mouse model, which could, if transferable to human patients, be a convenient alternative to the daily intake of gliptins (109).

Further research in developing alternatives toward Gliptins especially for long-acting medications would be an interesting new approach to improve lifestyle of patients.

#### Incretin-Based Therapies: Comparing DPP4i and GLP-1 Analogs

It is well accepted that incretin-based therapies are able to lower blood glucose levels and are therefore a treatment option for T2DM. There are mainly two approaches to target the incretin system (1) via inhibiting the enzymatic action of DPP4 and thereby upregulating GLP-1 levels physiologically and (2) via increasing GLP-1 levels pharmacologically. While GLP-1 receptor agonists (GLP-1-RA) directly target GLP-1, GLP-1-independent effects are also possible with the use of DPP4 inhibitors (DPP4i). These drugs might also affect the level of other DPP4 substrates and might therefore have a more complex mode of action.

However, there have been a lot of attempts to compare the effects of GLP-1-RA versus DPP4i in clinical studies. The results of these head-to-head comparisons are summarized in many current reviews (110–114). Most of these comparative studies agree that GLP-1 analogs are more effective in respect of glycemic control. Both incretin-based therapies are equally potent in lowering blood pressure and total cholesterol (110). Furthermore, both have the advantage of low incidence of hypoglycemia (110, 111). The results for body-weight lowering effects of DPP4i are heterogeneous throughout the studies (113), whereas beneficial effects on body-weight are well accepted for GLP-1-RA (110–112, 114). Therefore, some authors tend to prefer GLP-1-RA over the use of DPP4i (112). However, one should be aware of the fact, that GLP-1-RA have a higher incidence of gastrointestinal adverse events like nausea (110, 112, 114), which might be disadvantageous for elderly people who may be more prone to these side effects (114).

Furthermore, there are reports that DPP4i might also have cardioprotective effects (113), which will also shortly be discussed in section “Effect of DPP4 Inhibition on the Cardiovascular System” of this review. Despite the clear beneficial effects of incretin-based therapies, there are also concerns reported in respect of the risk for long-term complications like pancreatitis (115). These potential risks might, however, outweigh the benefits. These controversial discussions are well summarized by the reviews of Nauck and Butler (115, 116).

To really assess which medication is of more importance always depends on the special patient characteristic.

## Impact of DPP4 on T2DM-Relevant Organs and Associated Comorbidities

Dipeptidyl-peptidase 4 inhibitors exert glucose regulatory actions by prolonging the effects of GLP-1 and GIP, ultimately increasing glucose-mediated insulin secretion and suppressing glucagon secretion (117). Beside the glucose-lowering properties of DPP4 inhibitors, emerging evidence suggests that incretin-based therapies may also have a positive impact on inflammation, cardiovascular and hepatic health, sleep, and the central nervous system (118). However, the underlying mechanisms of these effects cannot be fully explained by lower blood glucose levels or increased GLP-1 bioavailability or signaling, and has to be further elucidated. Thus, the next section is focused on the role of DPP4 action in T2DM-relevant organs and associated comorbidities.

### Adipose tissue

Adipose tissue is the primary storage organ for excess energy. While the role of adipose tissue as a central source of energy has been recognized for centuries, in the past decade, it has become increasingly clear that adipose tissue also displays characteristics of an endocrine organ releasing a number of adipose tissue-specific factors, known as adipokines. During the progression of obesity, the ability of adipocytes to function as endocrine cells and to secrete multiple biologically active proteins is affected (119). Thus, adipose tissue has been shown to be a central driver of T2DM progression, establishing and maintaining a chronic state of low-level inflammation (120).

#### DPP4 Expression and Release Within Adipose Tissue

Recently, we showed that DPP4 is highly expressed in human primary adipocytes (4). Furthermore, DPP4 expression in adipose tissue is increased in obese compared to lean individuals in both subcutaneous and visceral adipose tissue (4, 5). Interestingly, visceral fat of obese patients exhibits the highest DPP4 level. According to the increased expression, we could identify sDPP4 as a novel adipokine released from primary human adipocytes. *In vitro*, the DPP4 release increased substantially during fat cell differentiation, and comparison with preadipocytes and adipose tissue, macrophages showed that adipocytes most likely represent the major source of DPP4 released from the intact organ to the circulation. Furthermore, the release of sDPP4 was elevated in adipose tissue explants of obese patients compared to lean controls and correlates with various classical markers of the metabolic syndrome, namely BMI, waist circumference, plasma triglycerides, and HOMA as an index of IR, as well as with fat cell volume and the adipokine leptin (4, 5).

How DPP4 expression affects adipocyte homeostasis can only be speculated. DPP4 might be involved in adipose tissue lipolysis. DPP4 recruits ADA, a monomeric enzyme catalyzing deamination of adenosine to inosine and ammonia (121, 122). It has been shown that DPP4-bound ADA has a 1000-fold greater activity than free ADA (123), which in turn may modulate the well-established antilipolytic effects of adenosine. Moreover, DPP4 is a strong inhibitor of the antilipolytic activity of NPY (76), which is one of the best peptide substrates of the enzyme (89). In this regard, Rosmaninho-Salgadoa and colleagues demonstrated that DPP4 stimulates lipid accumulation and PPAR-γ expression through cleavage of NPY suggesting that sDPP4 might stimulate adipocyte differentiation (77). However, it is noteworthy that the authors of this study were using tremendously high and non-physiological concentrations of sDPP4. On the contrary, a recent published study showed that DPP4 expression was strongly upregulated during adipocyte dedifferentiation *in vitro*. Hence, the authors concluded that DPP4 might be a major component in adipose tissue remodeling and cell plasticity (124). Nevertheless, enhanced abundance of DPP4 within adipose tissue of obese subjects may be involved in adipose tissue remodeling and substantially augments the lipolytic activity of enlarged adipocytes (57, 58).

Moreover, dendritic cells and macrophages resident in visceral adipose depots exhibit an increased DPP4 expression in response to inflammation or in the obese state (28). Since it is known that DPP4 exerts immunomodulating properties, Zhong et al. showed that membrane-bound DPP4 is co-localized with membrane-bound ADA on human dendritic cells resulting in an increased T-cell proliferation (28). Thus, it can be speculated that DPP4 might also play an important role in the chronic low-grade inflammation taking place in obesity and T2DM.

#### Adipose Tissue as Relevant Source of Circulating DPP4

Serum levels of sDPP4 are altered in many pathophysiologic conditions, including different types of cancer, allergic asthma, or hepatitis C (7). Our group was the first analyzing circulating sDPP4 in the context of obesity and the metabolic syndrome. DPP4 serum levels of morbidly obese men are elevated compared with lean controls and significantly correlated with BMI, the size of adipocytes in subcutaneous and visceral fat, and the adipocyte hormones adiponectin (negatively) and leptin. These data suggest that sDPP4 is related not only to increased body weight but also to other important parameters of adipose tissue physiology. In addition, sDPP4 release and serum concentration can be reversed to normal levels by surgery-induced weight loss (4). Thus, in obesity, both circulating levels of sDPP4 and sDPP4 release by adipose tissue are increased and correlate strongly with the metabolic syndrome but can be reduced to control levels by substantial weight loss. Thus, indicating that enlargement of visceral adipocytes in obesity may substantially contribute to the augmented level of circulating sDPP4 in obese patients.

#### Endocrine Effects of Soluble DPP4

Although there is clear evidence that increased circulating levels of sDPP4 are associated with hallmarks of obesity and type diabetes, such as whole-body IR, elevated BMI, and adipocyte hypertrophy, there are only few studies investigating the endocrine effects of sDPP4. We were the first showing that DPP4 consistently impairs insulin signaling at the level of Akt in primary human adipocytes (4). Enzymatic activity of sDPP4 appears to be involved in this process; however, since this work was done *in vitro*, it is most unlikely that the sDPP4-induced impairment of insulin action is due to an increased bioavailability of any DPP4 substrate. It might rather be that DPP4 inhibitors may also affect the binding properties of sDPP4 to its receptors, namely M6P/IGFII receptor (38) or PAR2 (39). For the latter, it is not only known that PAR2 signaling induces IR in adipocytes (125), but PAR2 might also be a substantial contributor to inflammatory and metabolic dysfunction (126). Although there is a hint that circulating sDPP4 itself might affect adipose tissue function, the exact mechanism has to be further investigated.

#### Impact of DPP4 Inhibition on Adipose Tissue

To further investigate the role of DPP4 in adipose tissue, several studies with DPP4 inhibitors were conducted. Interestingly, the administration of the DPP4-inhibitior des-fluoro-sitagliptin ameliorates linoleic acid-induced adipose tissue hypertrophy in β-cell-specific glucokinase haploinsufficient mice, a model of non-obese T2DM (127). Moreover, des-fluoro-sitagliptin protects against linoleic acid-induced adipose tissue inflammation illustrated by CD8^+^ T-cell infiltration. Due to the loss of GLP-1 receptors in adipose tissue, the authors exclude the involvement of GLP-1 and claim that the observed effects are due to the huge variety of DPP4 substrates. Thus, DPP4 inhibition might have pleiotropic effects in adipose tissue. A similar outcome has been observed in C57BL/6 mice fed a HFD. After linagliptin treatment, a significantly lower expression of the macrophage marker F4/80 was found compared with vehicle treatment. In line with these data, the authors demonstrated an increased insulin sensitivity after linagliptin treatment suggesting that DPP4 and adipose tissue inflammation play a pivotal role in the induction of IR. In 3T3-L1 cells, a murine predipocyte cell line, Rosmaninho-Salgado et al. demonstrated that the DPP4-inhibitor vildagliptin reduces lipid accumulation by inhibiting adipogenesis, without affecting lipolysis through NPY cleavage and subsequent NPY Y2 receptor activation (77).

With the recognition that adult humans also have BAT, an organ with substantial capacity to dissipate energy, BAT gained considerable interest as a novel target to treat or prevent obesity and its associated diseases. In 2013, the group around Shimasaki was the first reporting that des-fluoro-sitagliptin attenuated body adiposity, without affecting food intake, in C57BL/6 mice with diet-induced obesity (128). The increase in energy expenditure could be explained by enhanced levels of PPAR-α, PGC-1, and uncoupling protein-1 (UCP-1) in BAT as well as elevated levels of proopiomelanocortin in the hypothalamus. The beneficial effects of des-fluoro-sitagliptin on energy expenditure could only partly be ascribed to increased GLP-1 levels and have to be further validated. Shortly afterward, Fukuda-Tsuru et al. could confirm these data in the same animal model by administration of teneligliptin (129). Moreover, in this study, teneligliptin also reduces fat mass and suppresses HFD-induced adipocyte hypertrophy.

Collectively, there is clear evidence that DPP4 expression and release by adipose tissue play a key role in obesity and T2DM-associated processes, such as inflammation, adipocyte hypertrophy, and IR. However, the underlying mechanism of these beneficial effects is not fully understood and remains unclear in most of the publications.

### Pancreatic islets

β-cells play a central role in the etiology of T2DM. Due to failure of β-cell sensitivity to glucose and loss of β-cell mass, insulin secretion of these cells is not sufficient to counter balance IR, finally leading to T2DM. Although DPP4 inhibitors are now widely used for glycemic control, many debates are ongoing about their exact mode of action and their beneficial effects on pancreatic β cells.

#### Regulation of DPP4 Expression Within Pancreatic Islets

Interestingly, within the pancreatic islets, DPP4 localization differs between species. Islets of rodents showed a near-exclusive expression of DPP4 in β cells, with little expression in α-cells. In contrast, human and pig islets express DPP4 almost exclusively in α cells (130, 131). The species difference in the localization of DPP4 expression, and the possible physiological consequence of that difference, is unclear. Moreover, in a recent published study, it has been demonstrated that DPP4 activity was detectable in the conditioned medium of human islets suggesting that DPP4 is released from human islets as well (132). Under pathological conditions, islets of obese mice chronically fed a HFD that exhibit an increased DPP4 activity. The contrary was found in human islets from type 2 diabetic donors, showing a decreased DPP4 activity (131).

#### Impact of DPP4 Inhibition on Pancreatic Islets

Accumulating *in vitro* and pre-clinical data show that DPP4 inhibition has beneficial effects on T2DM induced β-cell dysfunction and apoptosis. Omar and colleagues demonstrated that DPP4 is not only present and active in mouse and human islets, but inhibition of islet DPP4 activity also has a direct stimulatory effect on insulin secretion, which is GLP-1 dependent (131). The same effect could be observed with a 2-week des-fluoro-sitagliptin treatment leading to increased insulin exocytosis by β cells from *db*/*db* diabetic mice (133). Furthermore, it could be shown that DPP4 inhibition is clearly associated with significantly increased β-cell mass and function in several models of T2DM (134–136). These beneficial effects were associated with the transcriptional activation of anti-apoptotic and pro-survival genes, as well as the suppression of pro-apoptotic genes in β cells (137). Additionally, Shah and collaborators showed that the DPP4-inhibitor linagliptin protects isolated human islets from gluco-, lipo-, and cytokine-toxicity (132). Accordingly, Akarte et al. reported anti-oxidative properties of vildagliptin shown by a dose-dependent decrease in nitric oxide concentrations in both serum and pancreatic homogenates of vildagliptin-treated diabetic rats (138).

Beside these pre-clinical and *in vitro* studies, only few are known about the beneficial effect of DPP4 inhibitors on β cells in human. In the short-term, 12-weeks vildagliptin treatment leads to a small increase in the capacity for insulin secretion (139). Treatment with vildagliptin over a longer period of time could also confirm an increased β-cell function in humans as a result of improved sensitivity of β cells to glucose (140, 141). However, this effect was not maintained after washout period, indicating that this increased capacity was not a disease modifying effect on beta cell mass and/or function. In the SAVOR-TIMI 53 trial, which was originally performed to assess the cardiovascular safety of saxagliptin, Leibowitz and colleagues recently reported that DPP4 inhibition may attenuate the progression of diabetes (142). This was evidenced by a decreased requirement for intensification of treatment associated with better preservation of glycemic control, as well as better sustained β-cell function as reflected in the fasting HOMA-2β during the 2-year follow-up period.

The exact mechanism how DPP4 inhibitors augment insulin secretion and increase β-cell mass *in vitro* and *in vivo* is still not fully understood, since not all these effects could be explained by elevated GLP-1 level or improved glycemic control associated with less glucotoxicity.

### Liver

Non-alcoholic fatty liver disease (NAFLD) describes a disorder with excessive deposition of fat within the liver with increasing prevalence in parallel to obesity and diabetes, which are major risk factors for NAFLD (143). Indeed, NAFLD is now the most common cause of chronic liver disease (144) and is present in one-quarter to one-half of diabetes patients (145). In the obese state, elevated triglyceride degradation in adipose tissue causes an increased hepatic uptake of fatty acids leading to fat accumulation within the tissue. Furthermore, reactive oxygen species (ROS), produced during lipid oxidation, are assumed to induce hepatocyte death and inflammatory reactions. Liver cirrhosis can be defined as the end stage of chronic liver diseases and is caused by progressive fibrosis. This process is characterized by excessive accumulation of ECM and activated hepatic stellate cells (146, 147) that ultimately results in nodular regeneration with loss of function (148).

#### Regulation of DPP4 Expression in the Liver

Although DPP4 exhibits a widespread organ distribution, the liver is one of the organs that highly expresses DPP4 (149). In the healthy human liver, intense staining for DPP4 was found in hepatic acinar zones 2 and 3, but not in zone 1. This heterogeneous lobular distribution suggests that DPP4 might be involved in the regulation of hepatic metabolism (150). Furthermore, mRNA expression levels of DPP4 were significantly increased in NAFLD livers compared to that in control livers (151). In accordance to that, DPP4 expression levels of NAFLD patients were negatively correlated with HOMA-IR and BMI, and positively correlated with total cholesterol levels, but not with ALT, lactate dehydrogenase (LDH), or triglyceride levels. Moreover, under conditions of high glucose, DPP4 expression was increased in HepG2 cells. However, other nutritional conditions, such as high insulin or the presence of fatty acids and cholesterol, did not affect DPP4 expression in these cells. Thus, the authors claim that enhanced DPP4 expression in NAFLD liver may rather be associated with IR than triglyceride accumulation and may promote the progression of liver disease via subsequent deteriorations in glucose metabolism. How increased DPP4 expression might affect liver function is still unknown. There are only a few hints that DPP4 might play a role in fibronectin-mediated interaction of hepatocytes with extracellular matrix (2, 36, 152). Beside DPP4 expression, there is only indirect evidence that hepatocytes also release DPP4 to the circulation, which will be further discussed in the next section.

#### Serum Level of DPP4 in Liver Disease

As previously discussed, hepatic DPP4 mRNA expression level in the livers is significantly higher in patients with NAFLD compared to healthy subjects (151). This upregulation of hepatic DPP4 expression is thought to be responsible for elevated DPP4 serum level in patients with liver disease (153–155). In line with this observation, serum DPP4 activity can be correlated with hepatic steatosis and NAFLD grading (156). Similarly, in patients with NAFLD, DPP4 activity in serum correlates with markers of liver damage, such as serum gamma-glutamyltranspeptidase and ALT levels, but do not correlate with fasting blood glucose levels and HbA1c values (156, 157). Thus, hepatic DPP4 expression in NAFLD may be directly associated with increased DPP4 serum level and may be involved in hepatic lipogenesis and liver injury.

#### Impact of DPP4 Inhibition on Liver Function

Since DPP4 inhibitors are widely used in clinical practice, this drug was also investigated as a potential new therapeutic strategy against the development of liver fibrosis and steatosis. Kaji and collaborators demonstrated that sitagliptin markedly inhibits liver fibrosis development in rats via suppression of hepatic stellate cell proliferation and collagen synthesis (158). These suppressive effects were associated with dephosphorylation of ERK1/2, p38, and Smad2/3 in the hepatic stellate cells. Additionally, hepatic steatosis could be prevented in several different animal models by DPP4 inhibition (127, 159, 160). Shirakawa and colleagues studied the effects of sitagliptin in glucokinase ± diabetic mice with diet-induced hepatic steatosis (127). Here, sitagliptin prevented fatty liver in both wild-type and glucokinase ± mice paralleled by decreased expression of sterol regulatory element-binding protein-1c, stearoyl-CoA desaturase-1, and fatty acid synthase, and increased expression of peroxisome proliferator-activated receptor-α in the liver. Furthermore, in a mouse model of non-alcoholic steatohepatitis, further studies indicated that linagliptin improves insulin sensitivity and hepatic steatosis in mice with diet-induced obesity (161) and ameliorates liver inflammation (162). The underlying mechanism of these beneficial effects has been further investigated by Ohyama et al. in *ob/ob* mice (163). The novel DPP4-inhibitor MK-0626 attenuates hepatic steatosis by enhancing AMPK activity, inhibiting hepatic lipogenic gene expression, increasing triglyceride secretion from liver, and elevating serum adiponectin levels.

Clinical data are very limited; however, several non-randomized trials conducted in small groups of diabetic patients demonstrated that DPP4 inhibitors improved the levels of liver transaminases and liver fat (164–166). Accordingly, Iwasaki et al. found a decrease in ballooning and non-alcoholic steatohepatitis scores in post-treatment liver biopsies (165, 166). Recently, in a comprehensive retrospective review of 459 type 2 diabetic patients, treated with DPP4-inhibitors, it was shown that DPP4 inhibitors improved the abnormality of the liver transaminases AST and ALT independent of HbA1c and body weight (167). Again in the majority of publications, the authors postulate that these beneficial actions were mediated through potentiation of direct GLP-1 actions on hepatocytes; however, it seems unlikely that hepatocytes express the canonical GLP-1 receptor (168).

In conclusion, accumulating studies indicate that DPP4 inhibitors are clinically useful for patients with T2DM accompanied by liver dysfunction based on fatty liver, and that DPP4 inhibition affects liver function regardless of diabetic status and obesity.

### Cardiovascular system

Cardiovascular complications (CVD) are common in patients with T2DM and a major cause of mortality (169). Atherosclerosis is the dominant cause of CVD and usually develops many years before any clinical symptoms are manifested. The underlying pathogenesis of atherosclerosis involves an imbalanced lipid metabolism and a maladaptive immune response entailing a chronic low-grade inflammation of the arterial wall. Endothelial cells and intimal smooth muscle cells represent the major cell types of the artery wall preserving vessel wall homeostasis. Together with leukocytes, they are the major players in the development of this disease. Beside atherosclerosis, T2DM also exacerbates heart failure associated with diastolic heart failure and coronary microangiopathy (170–172).

#### Regulation of DPP4 Expression and Release in Vascular Cells

Dipeptidyl-peptidase 4 is expressed in both microvascular endothelial cells of different human tissues, such as liver, spleen, lung, brain, heart (170, 172), and in human vascular smooth muscle cells (3). Under conditions of high glucose, DPP4 expression and activity were increased in human glomerular endothelial cells (173). Additionally, in STZ-induced diabetic rats, activity of membrane-bound DPP4 was increased, thereby reducing cardiac SDF-1 concentrations and causing impaired angiogenesis (174). Also hypoxia has been shown to regulate DPP4 expression in vascular cells. Regarding endothelial cells, there are conflicting data on the influence of hypoxia on DPP4 expression. In human microvascular endothelial cells as well as human umbilical vein endothelial cells, Eltzschig and colleagues showed that hypoxia increased DPP4 mRNA and protein level (175), whereas another study by Shigeta et al. observed a decreased protein level of DPP4 under hypoxic conditions in the same cells (174). However, in human vascular smooth muscle cells, we observed an increased DPP4 expression in response to hypoxia (3). In this particular study, we could also show that DPP4 is released from human vascular smooth muscle cells. However, only very little is known about the physiological role of the membrane-bound DPP4 within the vasculature. There is only one study showing that DPP4 forms a complex with ADA capable of degrading extracellular adenosine to inosine in endothelial cells. Increased inosine levels in turn are known to induce vasoconstriction due to mast cell degranulation (176).

#### Effect of DPP4 Inhibition on the Cardiovascular System

In several *in vitro* and pre-clinical studies, DPP4 inhibitors have been shown to exert important protective effects on the cardiovascular system. In this regard, it has been shown that DPP4 inhibitors decrease myocardial infarct size, stabilize the cardiac electrophysiological state during myocardial ischemia, reduce ischemia/reperfusion injury, and prevent left ventricular remodeling following MI (177, 178). Additionally, DPP4 inhibitors also exert vascular protective properties, including anti-inflammatory and anti-atherosclerotic effects and the ability to induce vascular relaxation (179, 180). To confirm cardiovascular safety or even protection of DPP4 inhibitors in humans, several cardiovascular outcome studies were conducted. However, several clinical trials, namely SAVOR-TIMI 53, EXAMINE, or VIVIDD in patients with established cardiovascular disease failed to confirm a cardio-protective effect (181–183). Even an increased cardiovascular risk for DPP4 inhibitors was discussed, since in the SAVOR-TIMI 53 trial, a significant increased hospitalization due to heart failure in the saxagliptin-treated group was observed. However, in the most recent published outcome study TECOS, the authors could show that among patients with T2DM and established cardiovascular disease, sitagliptin did not appear to increase the risk of major adverse cardiovascular events, hospitalization for heart failure, or other adverse events (184). As sDPP4 is an adipokine upregulated in obesity and T2DM that triggers IR and metabolic complications (4, 5), it might be speculated that the beneficial effects of DPP4 inhibitors would be higher in those early phases of the metabolic disorders previous to the development of established cardiovascular disease.

However, whether these beneficial effects observed in pre-clinical settings are due to increased levels of different DPP4 substrates or inhibition of direct effects of DPP4 remains unclear and will be assessed in more detail in the following section.

#### DPP4 Substrates: GLP-1 Dependent Effects of DPP4 Inhibitors

Since several studies have identified a role for GLP-1 receptor (GLP-1R) signaling in DPP4-dependent cardioprotection, it is suggested that GLP-1 itself has favorable cardiovascular effects. Indeed, mRNA transcripts of the GLP-1R have been detected in the heart of rodents (185, 186) and humans (187). Furthermore, GLP-1R has also been localized to mouse aortic smooth muscle and endothelial cells, as well as monocytes and macrophages (188).

Regarding MI and heart failure, pre-clinical studies have demonstrated that DPP4-deficient rats subjected to 45 min of ischemia with 2 h or reperfusion exhibited cardioprotection illustrated by reduced infarct size, improved cardiac performance, and reduced levels of BNP compared to control rats (189). These beneficial effects could be partially reversed by co-administration of the GLP-1R antagonist exendin (9–39). Accordingly, administration of exendin (9–39) reversed the sitagliptin-induced improvement in ventricular function in Sprague Dawley rats with transient cardiac ischemia (190). Additionally, in a rat model of chronic heart failure, GLP-1 analogs were able to improve cardiac function and morphology, with a concomitant amelioration of hyperglycemia and hyperinsulinemia (191).

Regarding the vascular system, continuous infusion of the GLP-1 analog exendin-4 reduced monocyte adhesion to aortic endothelial cells, associated with a reduction in atherosclerotic lesion size in non-diabetic C57BL/6 and *ApoE*^−/−^ mice. Furthermore, treatment for 1 h with exendin-4 reduced the expression of the pro-inflammatory cytokines, TNFα and MCP-1, in response to lipopolysaccharide (LPS) (188). In addition, exendin-4 stimulates proliferation of human coronary artery endothelial cells through endothelial nitric oxide synthase (eNOS)-, protein kinase A (PKA)-, and PI3K/Akt-dependent pathways (192, 193). Accordingly, in humans, preliminary data confirm the ability of GLP-1 to protect from high glucose-induced endothelial dysfunction in the post-meal phase (194). In a model of vascular injury, it has been shown that continuous infusion of exendin-4 reduces neointimal formation at 4 weeks after injury without altering body weight or various metabolic parameters (195). From *in vitro* studies, Goto et al. suggest that this effect was mediated by the ability of GLP-1 to suppress platelet-derived growth factor (PDGF)-induced proliferation of vascular smooth muscle cells. In contrast, in a pre-clinical study, combining HFD and STZ treatment in ApoE^−/−^ failed to detect evidence for GLP-1R-dependent reduction of lesion size in the thoracic or abdominal aorta (168). The authors discuss that the duration of treatment, the dose of the GLP-1 agonist, or the age of mice might be responsible for the lack of anti-atherogenic activity in this study.

However, in patients with heart failure, pilot studies also suggest cardioprotection by GLP-1 infusion (196, 197). Accordingly, a large retrospective analysis indicates that patients treated with the GLP-1 analog exenatide had a significant 20% reduction of CVD events compared with patients on other glucose-lowering agents (198). Nevertheless, studies showing cardiovascular protective effects of GLP-1 were carried out using either native GLP-1 or recombinant GLP-1 analogs at high concentrations or in a way that induced supraphysiological GLP-1 signaling. Considering that DPP4 inhibition restores GLP-1 signaling within the physiological range, beneficial effects of DPP4 inhibitors might be different to those of GLP-1 analogs.

#### DPP4 Substrates: SDF-1- and BNP-Dependent Effects of DPP4 Inhibitors

But beside GLP-1, there are further substrates of DPP4, which might play a role in the favorable cardiovascular effects of DPP4 inhibitors. Two of the most promising candidates are SDF-1α and brain natriuretic peptide (BNP). As already mentioned in section “Stromal Cell-Derived Factor-1α/CXCL12,” SDF-1 is a chemokine promoting stem-cell homing of EPCs by binding to its receptor C–X–C motif chemokine receptor type 4 (CXCR4). EPCs are derived from the bone marrow and are known to promote vascular repair and neoangiogenesis. When vascular damage occurs, local growth factors and cytokines signal the bone marrow to release EPC targeted to the injured sites. EPC then differentiate into mature endothelial cells and assist in the reconstruction of the vasculature (199). In mice, genetic deletion or pharmacologic inhibition of DPP4 is able to increase the homing of CXCR4+ EPC at sites of myocardial damage, resulting in a reduced cardiac remodeling and improved heart function and survival (200). In a human study, Fadini et al. demonstrated that type 2 diabetic patients receiving a 4-week course of therapy with the DPP4-inhibitor sitagliptin show increased SDF-1α plasma concentrations and circulating EPC levels (199). Additionally, SDF-1 engineered to be resistant to DPP4 cleavage, and delivered by nanofibers, improves blood flow in a model of peripheral artery disease (201). Collectively, these studies implicate a rationale to use DPP4 inhibitors for vascular repair through stimulation of EPC and neovascularization.

Brain natriuretic peptide, another substrate of DPP4, plays an important role in regulating body fluid homeostasis and vascular tone through binding and subsequent activation of the cGMP-coupled natriuretic peptide receptor type A (NPR-A) (202). BNP is secreted predominantly by ventricular cardiomyocytes in response to increased wall stress. Thus, elevated BNP is a sensitive marker of heart failure and appears to play a role in cardiac remodeling and healing after acute MI (203–205). DPP4 cleavage of the physiologically active BNP (1–32) to BNP (3–32) effectively lowers plasma cGMP levels, reduces diuresis and natriuresis, and inhibits vasodilatation (83, 202).

#### Endocrine Effects of Soluble DPP4 on Cardiovascular Homeostasis

Although it is well established that serum levels of sDPP4 are altered in several pathological conditions and that sDPP4 is released from vascular cells, only a minor part of research has focused on potential endocrine effects of this proteolytic enzyme.

Considering that DPP4 is discussed in immunomodulation, it might be speculated that the inhibition of DPP4 modulates responses occurring within early or late atherosclerotic lesions. In low-density lipoprotein receptor-deficient (LDLR^−/−^) mice, Shah et al. could demonstrate that exogenously injected DPP4 increases monocyte migration *in vivo* (180). Although these pro-migratory properties of DPP4 could be completely inhibited by sitagliptin, the underlying mechanism of these effects remains unclear. Moreover, the combined treatment of sDPP4 and LPS leads to increased expression and secretion of the pro-inflammatory cytokines, TNFα and IL-6. This upregulation was achieved by elevated levels of ERK, c-Fos, NF-κB p65, NF-κB p50, and CUX1, all factors known to bind to the promotor of TNFα and IL-6 (180). In accordance to that, Ikushima and collaborators observed that sDPP4 binds to the M6P/IGF-IIR resulting in enhanced transendothelial T cell migration (206). In a further study, sDPP4 binding to M6P/IGF-IIR leads to elevated ROS levels in HUVECs. In both studies, binding of DPP4 to this particular receptor was completely prevented by a DPP4 inhibitor (207).

In human vascular smooth muscle cells, we could show that sDPP4 activates the MAPK and NF-κB signaling cascade resulting in pro-atherogenic changes in human vascular smooth muscle cells illustrated by an increased proliferation, the induction of iNOS and elevated expression, and secretion of pro-inflammatory cytokines (39). Additionally, we observed that all these detrimental effects of sDPP4 were PAR2 mediated, since both a PAR2 antagonist and PAR2 silencing completely prevented the sDPP4-induced effects. In collaboration with the group of Sánchez-Ferrer, we further showed that sDPP4 exhibits direct effects on vascular function illustrated by vascular reactivity of murine mesenteric arteries (208). sDPP4 impaired the endothelium-dependent relaxation to acetylcholine in a concentration-dependent manner by up to 75%, without modifying endothelium-independent relaxation to sodium nitroprusside. Again, enzymatic activity of DPP4 appears to be involved in this process. Similarly, the cyclooxygenase inhibitor indomethacin and the thromboxane A2 receptor antagonist SQ29548 abrogated the impairing action of DPP4. These data suggest that DPP4 directly impairs endothelium-dependent relaxation through a mechanism that involves cyclooxygenase activation, and likely the release of a vasoconstrictor prostanoid. Since sDPP4 has been reported not only to contribute to monocyte migration and macrophage-mediated inflammatory reactions but also stimulates the proliferation of human coronary artery smooth muscle cells as well as impairs endothelium-dependent vasorelaxation, it might be speculated that sDPP4 itself acts as a risk factor for atherosclerosis.

**Figure 2:**
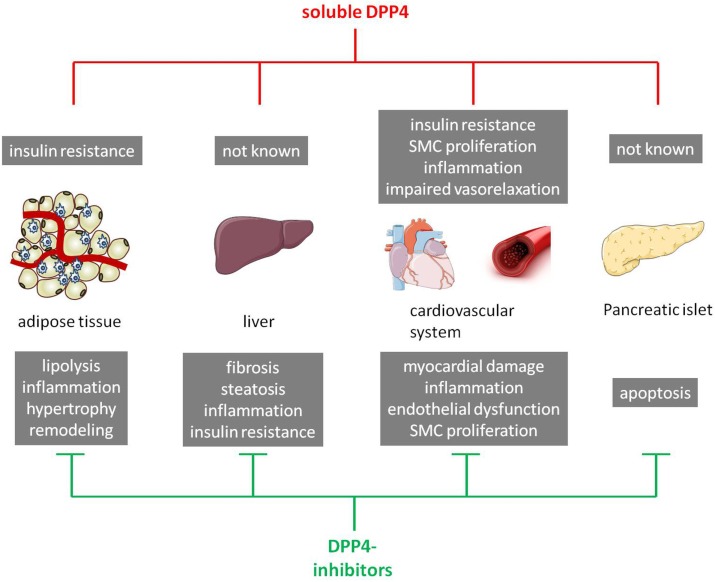
**Schematic overview of the impact of soluble DPP4 and DPP4 inhibitors on T2DM-relevant organs/tissues**. In the upper panel, direct effects of soluble DPP4 (in red) on different organs/tissues are presented (gray boxes). The lower panel shows known effects of DPP4 inhibitors (in green) in these particular organs/tissues (gray boxes). SMC, smooth muscle cells.

Collectively, this section emphasizes that both membrane-bound and sDPP4 and its inhibition are not only playing an important role in glucose homeostasis but also in several other processes and organs involved in the pathogenesis of T2DM (Figure 2). This supports the notion that DPP4 exhibits pleiotropic properties that are not fully understood so far and have to be further elucidated in the future.

## Conclusion

Dipeptidyl-peptidase 4, originally identified as an enzyme nearly 50 years ago, has now been recognized to exert pleiotropic functions with substantial impact for a variety of diseases. The complexity of DPP4 action stems from (i) a long list of substrates cleaved by the enzyme including hormones, growth factors, and cytokines, (ii) an additional function of this protein being a binding partner at the surface of different cells, specifically immune cells, and (iii) the recent discovery that DPP4 is an adipokine with different endocrine functions. Thus, an integrated view on this molecule is required to more precisely understand its impact for metabolic diseases like type 2 diabetes. For this disease, DPP4 inhibition has gained substantial interest, mostly related to the DPP4 substrate, GLP-1. As shown here, other substrates like SDF-1 and BNP should also be taken into account and may help to better understand the therapeutic potential of DPP4 inhibitors. In this context, the direct effects of DPP4 inhibitors require to be assessed in more detail, and several aspects like the cardio-protective function of DPP4 inhibition remains controversial. Finally, soluble DPP4 is emerging as a new research line, putting this molecule to the list of adipo-cytokines with pro-inflammatory and proliferative function. Combining the accumulated knowledge on DPP4 will lead to an improved understanding of its impact for health and disease.

## Conflict of Interest Statement

The authors declare that the research was conducted in the absence of any commercial or financial relationships that could be construed as a potential conflict of interest.
